# Data-driven analysis of facial thermal responses and multimodal physiological consistency among subjects

**DOI:** 10.1038/s41598-021-91578-5

**Published:** 2021-06-08

**Authors:** Saurabh Sonkusare, Michael Breakspear, Tianji Pang, Vinh Thai Nguyen, Sascha Frydman, Christine Cong Guo, Matthew J. Aburn

**Affiliations:** 1 QIMR Berghofer Medical Research Institute, Brisbane, QLD 4006 Australia; 2 School of Medicine, The University of Queensland, Brisbane, Australia; 3 Northwestern Polytechnical University, Xi’an, Shaanxi China; 4 The University of Newcastle, University Drive, Callaghan, NSW 2308 Australia

**Keywords:** Neuroscience, Peripheral nervous system, Autonomic nervous system, Human behaviour

## Abstract

Facial infra-red imaging (IRI) is a contact-free technique complimenting the traditional psychophysiological measures to characterize physiological profile. However, its full potential in affective research is arguably unmet due to the analytical challenges it poses. Here we acquired facial IRI data, facial expressions and traditional physiological recordings (heart rate and skin conductance) from healthy human subjects whilst they viewed a 20-min-long unedited emotional movie. We present a novel application of motion correction and the results of spatial independent component analysis of the thermal data. Three distinct spatial components are recovered associated with the nose, the cheeks and respiration. We first benchmark this methodology against a traditional nose-tip region-of-interest based technique showing an expected similarity of signals extracted by these methods. We then show significant correlation of all the physiological responses across subjects, including the thermal signals, suggesting common dynamic shifts in emotional state induced by the movie. In sum, this study introduces an innovative approach to analyse facial IRI data and highlights the potential of thermal imaging to robustly capture emotion-related changes induced by ecological stimuli.

## Introduction

Galvanic skin response (GSR) and heart rate (HR) metrics are commonly employed in psychophysiological studies^1–3^. However, it has been argued that measures targeting a variety of somatic effects may be best able to capture emotion-related brain-body states^2^. An innovative technique which compliments the traditional measures is facial infra-red imaging (IRI). IRI quantifies the temperature fluctuations of the face, predominantly resulting from blood flow changes^4^ although it has also been shown to capture the temperature effect of perspiration^5^. As a contact-free technique, it allows ecologically valid experimental conditions which recent studies have exploited^6^. Although it has not yet been as widely used as GSR and HR measures, thermal imaging has shown promising results in affective research^7–9^.

The current use of facial thermal imaging analysis is based on ad hoc choices of facial regions, usually chosen a priori and used with a region of interest (ROI) analysis i.e. extracting the thermal signals from specific facial regions like the nose-tip, the cheeks and the forehead^7–12^. ROI-based thermal signal extraction can be strongly influenced by motion artefacts^13^ with only a few studies implementing motion tracking or motion correction^14,15^. Furthermore, ROI based approaches have their own unique challenges and limitations. First, they only exploit a small portion of the information encoded in the whole face. Second, manual steps are time consuming and only feasible for the analysis of small data sets with low sampling rate. While several useful improvements in methods have been proposed such as in tracking^16^, displacement correction of frames by matching anatomical landmarks^17^ and analysis based on machine learning^18^, these have yet to be adopted in general practice. Lastly, different *apriori* choices for the size of ROIs, their shapes and placement can further introduce biases^4^.

Although thermal signal extraction based on ROIs has provided many insights into thermal effects in affective research, these methods can be complemented by data-driven approaches which uncover underlying data features with less *apriori* assumptions. Independent component analysis (ICA), specifically spatial ICA (sICA), is a subset of such methods for blind signal separation employed under assumptions of statistical independence of the source signals^19^. Due to the effectiveness in capturing the essential structure of diverse kinds of data, ICA has been widely used in many applications, such as functional magnetic resonance imaging (fMRI) analysis^20,21^ which measures the haemodynamic changes as a proxy for neuronal activity. Similar to the brain, facial skin has an extensive distribution of blood vessels. Furthermore, temperature changes associated with the face can have various contributions, including stimulus induced changes but also respiratory confounds, perspiration, and noise. The diverse nature of these signals suggests that blind signal separation techniques could be advantageous for isolating the different sources of signal^19^ and distinct spatial components.

With these considerations in mind, we sought to employ an sICA approach to characterize the dynamic facial thermal responses during naturalistic emotional experience. For this we acquired facial IRI data alongside other traditional physiological measures while subjects watched a 20-min emotional movie. Naturalistic stimuli have emerged as an alternative to strictly controlled paradigms and stimuli, such as pictures and sounds, as they offer better ecological validity^22,23^ and induce robust physiological responses^24^. Furthermore, movies have been previously used in a thermal imaging study to induce emotions^25^. To analyse thermal signals in a maximally naturalistic setting where participants were free to move, we combined state-of-the-art motion correction method based on optical flow with sICA to extract dynamic temperature changes that manifest in different facial regions. Since ROI-based facial IRI studies have recognized the nose-tip as the most sensitive region for temperature fluctuations, we hypothesised that a nose-tip spatial component would be reliably captured with the sICA method as well.

The secondary aim of this study was to test the shared nature of physiological responses between subjects. For the three main modalities GSR, HR and thermal imaging we aimed to test whether these measures show consistency of the dynamic responses across subjects. In neuroimaging studies using naturalistic stimuli such as movies, inter-subject correlation (ISC) analyses have demonstrated common covarying patterns of brain activity^22,26,27^. Here we leverage this analytical technique to investigate whether subjects share a common temporal response in these physiological signals (heart rate metrics, skin conductance and thermal responses).

## Materials and methods

### Thermal imaging and physiological data acquisition

Twenty healthy human participants (11 females, aged 22–30 years, mean = 25.7 years) were recruited for the study. Informed consent was obtained from all participants. All participants had normal or corrected-to-normal vision (using contact lenses if applicable). Exclusion criteria included habitual smoking, the presence of a chronic illness (e.g., cardiovascular or thyroid conditions), psychological disorders (e.g. depression or anxiety) or any other illness requiring regular medication. The study was approved by the Research Ethics Board of QIMR Berghofer and performed in agreement with the Declaration of Helsinki. The participants had the choice to withdraw from the study at any time. Each participant was compensated with a $50 voucher for their time. At the end of the data acquisition session a questionnaire was completed by the participants regarding subjective ratings of emotional response to the movie (Table S1). Physiological data (thermal, GSR and HR) from three participants were excluded from analysis due to the failure of accurate trigger information and incomplete data acquisition. Questionnaire ratings could not be recorded from one subject. Informed consent to publish identifying images (RGB and thermal) was obtained from two subjects.

Prior to the data acquisition, participants acclimatized for about 10 min within the experimental room. We adopted this practice as per prior studies^7,11^ and recommendations suggested^4^. The temperature and the humidity of the room were kept within a steady range (22 ± 2 °C; 55–65% relative humidity). Where necessary, participants’ hair was secured away from their forehead with an unobtrusive hat. Participants were also asked to avoid alcohol and caffeine for at least 2 h prior to the experimental session to minimize the vasoactive effects that these substances have on the skin temperature. Testing was performed exclusively between 2 and 5 p.m. to avoid any potential confounding effects of the circadian rhythm. After the participants had assumed a comfortable posture in a fixed chair, GSR and electrocardiograph (ECG) electrodes were attached to fingers and arms, respectively. A thermal imaging camera and an RGB video camera were then manually focused on the subject’s face. The researcher checked the recording quality and left the experimental room but retained audio-visual contact via CCTV. The paradigm consisted of a ~ 20-min short movie The Butterfly Circus^28^. This stimulus has been used in our prior study and was selected by a film critic^26^ because of its critical acclaim and its ability to evoke an emotional experience. Ten seconds of countdown was prefixed to the movie. Stimuli were presented on a 24-inch computer screen 40 cm in front of the subject. The sound was kept to the same levels for all subjects and was presented via two loudspeakers placed beside the stimulus screen.

An in-house built integrated hardware and software experimentation platform LabNeuro was used to integrate the multi-modal data acquisition. Thermal imaging of the face was performed by a FLIR A615 camera with a 15 mm lens, an uncooled Vanadium Oxide (VoX) detector producing images of 640 × 480 pixels in size placed approximately 100 cm away from the participants’ faces. The FLIR A615 provides a temperature detection range from − 20 to 2000 ℃ with the NEDT (noise equivalent differential temperature) smaller than 0.05 ℃ at 30 ℃. The thermal camera response was blackbody-calibrated to nullify noise-effects related to the sensor drift/shift dynamics and optical artifacts. For this camera emissivity correction was configurable from 0.01 to 1.0 and was set at 0.98. The sampling rate for thermal imaging was set at 5 Hz. This was performed in order to generate sufficient frames to balance out any potential movement artifacts by the participant. The cameras were incorporated into LabNeuro using the LabVIEW Image Acquisition library.

GSR recordings were obtained by two Ag/AgCl electrodes (0.8-cm diameter) filled with a conductive paste and attached to the distal phalanges of the index and ring fingers of the participant’s left hand. Skin conductance was recorded using a standard constant voltage system of 0.5 V and recordings were continuously digitized by an A/D converter with a sampling rate of 2 kHz. The recording of GSR data was acquired using National Instruments (NI) CompactDAQ modular IO hardware and software for the system was written using NI LabVIEW and NI Biomedical Toolkit. For ECG data collection, electrodes were attached to the mid-upper left arm, left wrist, and mid-upper right arm. We asked participants to put their hands-on chair hand-rests to minimize motion artifacts. ECG data was recorded with a sampling rate of 2 kHz. GSR signals and ECG data were collected simultaneously with the thermal imaging data.

### Extraction of facial thermal responses

**Figure 1:**
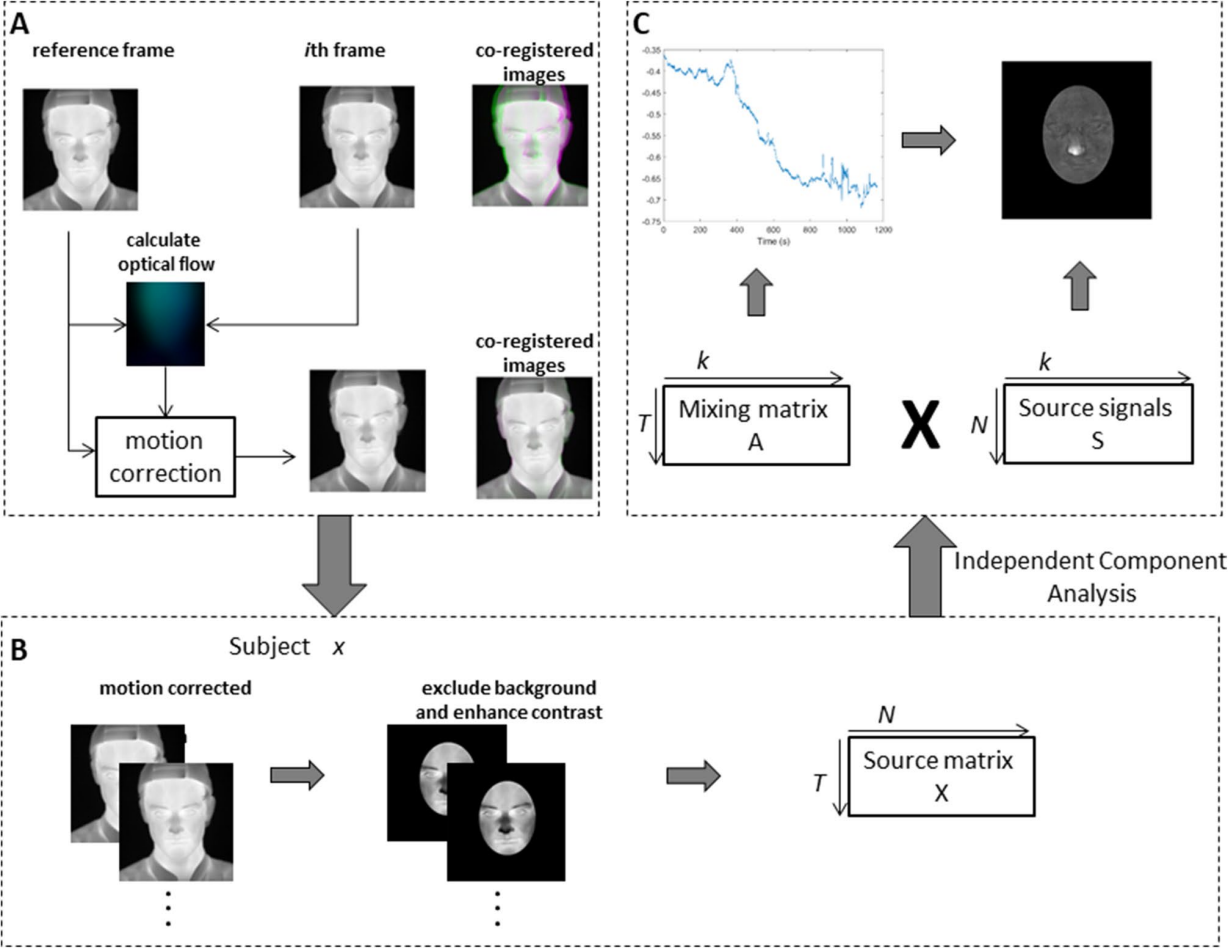
The computational pipeline for employing spatial independent component analysis (sICA) on thermal imaging data (**A**) Motion correction framework showing co-registered images with and without application of optical flow. For each subject, the whole thermal imaging data are aggregated into a single matrix, in which each row represents the thermal imaging data in one time point and each column stands for the time series of one single pixel (**B**) A mask to exclude background, i.e. neck and clothes, and retain only the face was applied to each frame. The data from these images were used as the source matrix (**C**) sICA—illustration of the mixing matrix, each column of which represents the time course of the corresponding source signal. An exemplar time series of nose components is shown. Each row of the source signal matrix represents one independent spatial map. Thermal image of subject L06 is used for illustrative purposes.

The pipeline for this workflow is illustrated in Fig. 1. First the thermal frames were pre-processed to eliminate motion by applying a dense optical flow algorithm^29^. Specifically, the nonlinear motion vector field between each frame pair was first estimated by this method. Discontinuities in the transformation, which could distort the boundary of the face, were effectively removed by iterating motion correction and a Gaussian smoothing step applied to the motion vector field until convergence (where the displacement of all pixels is less than 1).

Similar to its use in fMRI data, spatial ICA was then applied to remove artifacts and to unmix statistically independent (true) sources of facial thermal signal change. To reduce noise and the number of parameters to be estimated, pre-whitening and dimension reduction were employed before the application of ICA. sICA was applied using a publicly available software package FastICA (http://research.ics.aalto.fi/ica/fastica/code/dlcode.shtml)^30,31^. The specific parameters inputted in the code are provided in supplementary Table S3. The complete pipeline of the implementation of ICA together with motion correction is shown in Fig. 1.

Motion correction is a separate pre-processing step before sICA is employed. It is the first step (Fig. 1A) and does not depend on any ROI or facial components. It eliminates head and face motion by non-linearly morphing each frame back to coincide with preceding frames, leaving a video where the head does not move in space or rotate. This motion correction is important for sICA because the ICA model assumes that changes in a pixel’s intensity represent changes in temperature and not underlying movement of the face. The novel pre-processing method to remove motion is also valuable in combination with a traditional ROI signal extraction, as it serves to eliminate the issue of motion artifacts. In the research field of computer vision, this general problem of optical flow motion correction is a well-researched problem, so we are here bringing its novel application to psychophysics.

Though the motion correction and the ICA are both automated processes, the selection of physiologically meaningful independent components still needs manual inspection. To identify independent components with the most anatomico-physiological meaning we restricted attention to spatially localized components corresponding to broad anatomical boundaries such as nose, cheeks and forehead. The time series corresponding to each component was then normalized to have values between 0 and 1.

### Comparison of nose component signal with region-of-interest based nose-tip signal

For validation and comparison of sICA against an ROI approach, a circle of 9-pixel radius (area ~ 250 pixels) was used to define an ROI to extract thermal signals from the nose-tip of the thermal data (Fig. 4A left). An identical size of ROI was chosen to have uniformity across participants irrespective of face morphology. The same motion correction was used with the ROI and sICA to allow comparison. The time series extracted by ROI were normalized to have values between 0 and 1 for each subject to mitigate against spurious inter-subject differences. The correlation of ROI based nose-tip signal to that obtained from nose IC was computed, and significance of this correlation assessed using a non-parametric permutation test. Specifically, we calculated the group mean of the Pearson correlation between each subject’s nose component signal and their ROI based nose-tip signal. To generate the null distribution, each permutation used the ROI nose-tip signal with a random circular shift, to preserve temporal autocorrelation, and the group mean of the correlation between these shifted data and the thermal signals was computed. The null distribution was generated from 5000 permutation realizations.

### Heart rate variability

The ECG signal was pre-processed using QRSTool software^32^ to detect the R peaks with the ability to manually correct for missed peaks. Inter-beat interval (IBI) time series was then computed from this and normalised to have values between 0 and 1. R peak data were further analysed using HRVAS toolbox^33^ to obtain heart rate variability (HRV) frequency domain measures. These were calculated via the auto-regressive method using a window size of 16 s, with 15 samples overlap, nfft of 1024 and cubic spline interpolation rate of 2 Hz. These parameters were chosen as they had previously been demonstrated to capture robust responses to simple stimuli even with a short duration of data^23^. HRV data metrics were computed for the whole stimulus but edge effects for frequency estimation led to loss of approximately 8 s of data at the beginning and the end. Time–frequency decompositions of inter-beat intervals are typically linked to autonomic influences in distinct frequency bands. Lower frequency (LF) HRV (0.04–0.14 Hz) mainly reflects changes in sympathetic and parasympathetic outflows, while high frequency (HF) variability (0.15 to 0.4 Hz) is primarily due to modulation of parasympathetic outflow^34,35^. However, the interpretation of low frequency HRV has been questioned^36,37^. Respiratory sinus arrhythmia (RSA) is a major contributor to HF HRV and is thought to be due to respiration modulating the cardiac vagal activity^38^.

### Comparison of nose component signal with skin conductance (GSR)

GSR is a standard measure of arousal. Event based studies have found an inverse relation between GSR and nasal temperature^15,39^. The GSR time courses were first detrended and low pass filtered at 5 Hz and subsequently normalized to have values between 0 and 1 for each subject to mitigate against spurious inter-subject differences in baseline. For statistically comparing GSR with nose component signal, identical permutation testing procedure as used for comparison of thermal signals to that ROI based signal was used.

### Inter-subject correlation of physiological signals

Pearson correlation coefficients between each pair of participants were computed separately for both thermal signals, GSR data and heart rate data. Inter-subject correlation (ISC) was computed as the mean pairwise correlation between participants. With 17 participants, each analysis comprised a total of 136 correlation pairs. Non-parametric permutation tests were used to identify statistically significant inter-subject correlation (*p* = 0.05)^40,41^. Specifically, the null distribution was generated from 5000 permutation realizations. These permutations comprised circularly shifted data in a way to preserve temporal autocorrelation in the physiological signals but disrupt correlations between subjects.

### Testing for similarity between two different recordings at group level

To assess statistically whether two different physiological responses were significantly correlated (such as the nose IC and nose-tip ROI thermal signals, the nose IC thermal signals with GSR, and the nose IC thermal signals with movie emotions), we employed non-parametric permutation testing. We first calculated for each subject the Pearson correlation coefficient between the two signals of interest. The correlation values were then averaged for all the subjects to obtain a group mean correlation value. To statistically test the significance of the obtained group mean correlation value, we applied non-parametric permutation tests with a null model where data from one modality was randomly circularly shifted relative to the other (thus preserving autocorrelation), before computing the group mean correlation of the null. Five thousand realizations were used to generate the null distribution.

### Facial expression analysis

Facial emotions expression scores, both for the movie actors and for the subject’s responses were obtained by processing the movie frames, and the RGB video recordings of the each subject through the Microsoft CNTK Face API (Cognitive Toolkit)^42^. This software has been rated as one of the best for emotion categorisation^43^. The software takes a facial image as an input and returns the likelihood of each emotion class, across a pre-specified set of emotions, for each face in the image. The emotion classes permissible are anger, contempt, disgust, fear, happiness, neutral, sadness and surprise. They are ranked in descending order, with the overall total score summing to 1. The score was averaged for all the frames and averaged for each second. Briefly, emotion recognition is usually based on three key steps: (1) face detection, (2) feature detection—such as eyes and eye corners, brows, mouth corners, the nose-tip etc. and (3) feature classification—translation of the features into Action Unit codes, emotional states, and other affective metrics^44^. We summed the emotion scores for each emotion category for each subject before undertaking inter-subject correlation analysis to investigate the consistency of facial expressions among subjects.

## Results

Subjective ratings (SI Table S1, Fig. S1) showed that the participants perceived the movie to be positively valenced (Table S1 Question 1: mean = 5.95, SD = 1.43) as well as emotionally intense (Table S1 Question 2: mean = 5.53, SD = 1.26).

### Consistent facial components identified by sICA

**Figure 2:**
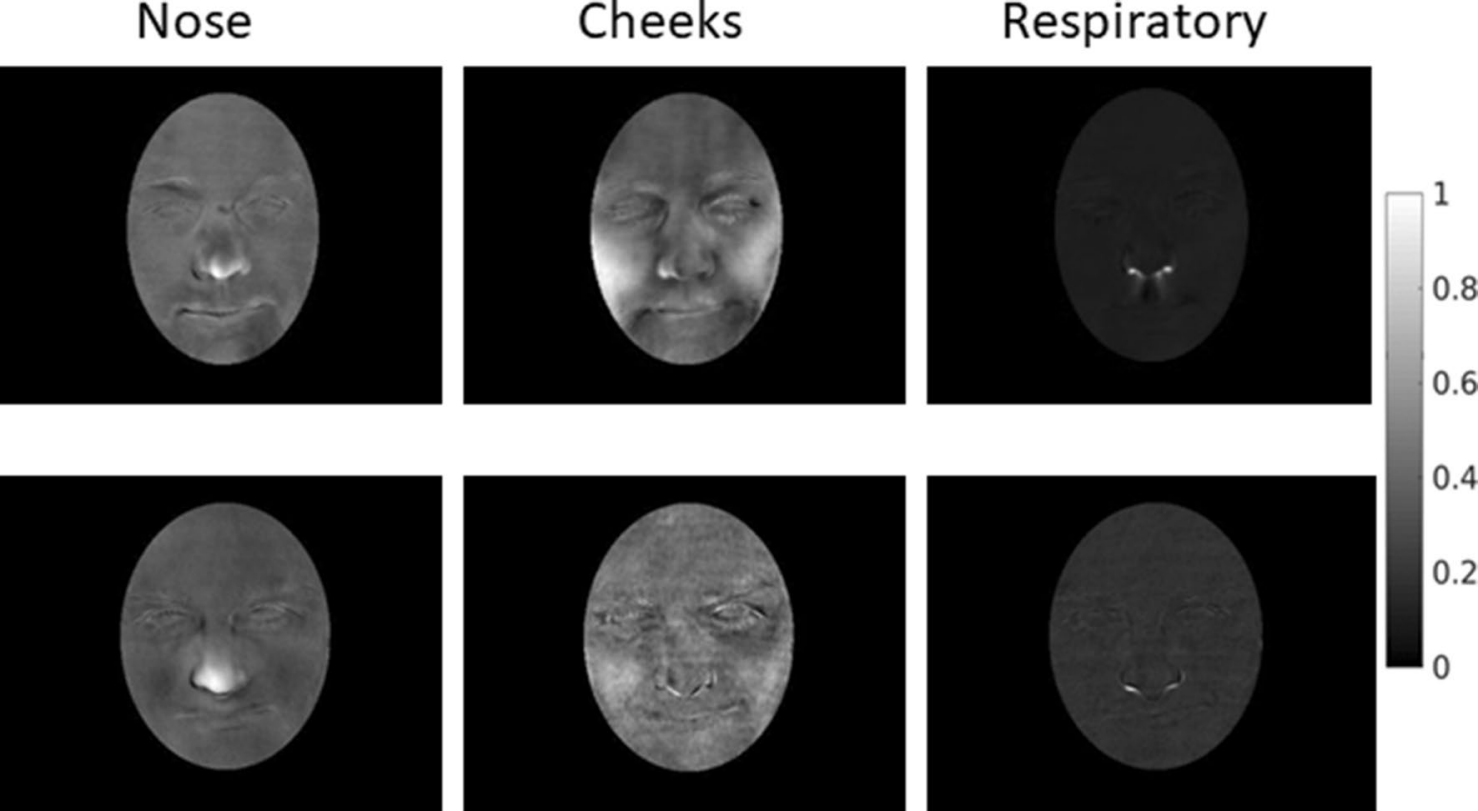
Distinct spatial components. Representative components from one subject (L08) are shown. Three components were consistently identified in all subjects (except respiratory component absent in one subject). Color scale normalized between 0 and 1 for each component for display. Results from two subjects are shown here.

We extracted fifty independent components (ICs) from each subject. Of these, three components could be consistently identified across all subjects based on spatial and temporal characteristics. These were a nose component which mainly included pixels on the nose-tip, a cheek component which mainly contained pixels distributed on bilateral cheeks, and a respiratory component. The spatial patterns of these components clearly follow anatomical boundaries of facial features, supporting that they are driven by underlying physiological processes (Fig. 2).

**Figure 3:**
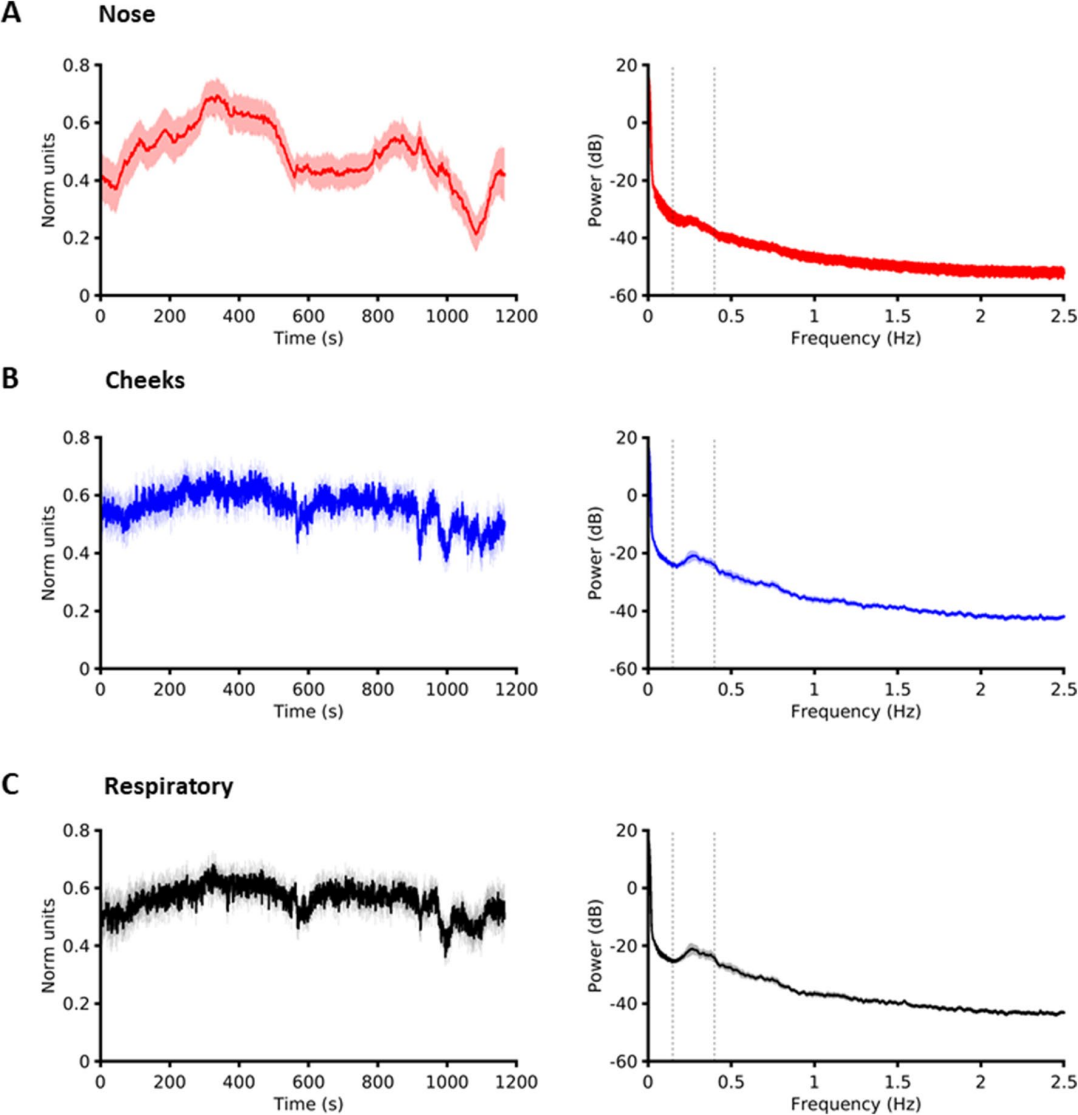
Group averaged component signals and their spectral signatures. (**A**) nose component signal and its power spectra (right). (**B**) bilateral cheek component signal and its power spectra (right). (**C**) respiratory component signal and its power spectra (right). Vertical dashed lines on the spectral plots indicates the normal respiratory frequency range of 0.16–0.35 Hz. Respiratory and cheek components both seem to be affected by respiration whereas the nose component seems minimally affected by it. Shading indicates SEM.

The temporal courses of these three ICs showed gradual evolution over the course of the movie (Fig. 3 left). To investigate whether the nose and the cheek ICs identified were distinct from the respiratory component, we compared the power spectra of these signals (Fig. 3 right). A frequency range of 0.16–0.35 Hz is indicative of normal respiration frequency^45^. The nose IC was minimally affected by respiration whereas the power spectra of both the cheek and the respiration IC exhibited a peak at frequency range of 0.16–0.35 Hz. The similar time courses and power spectra of the bilateral cheeks to that of the respiration IC is consistent with the cheek IC’s diffuse spatial spread which likely includes respiration affected signals.

### Validation of sICA nose component by ROI tracking method

**Figure 4:**
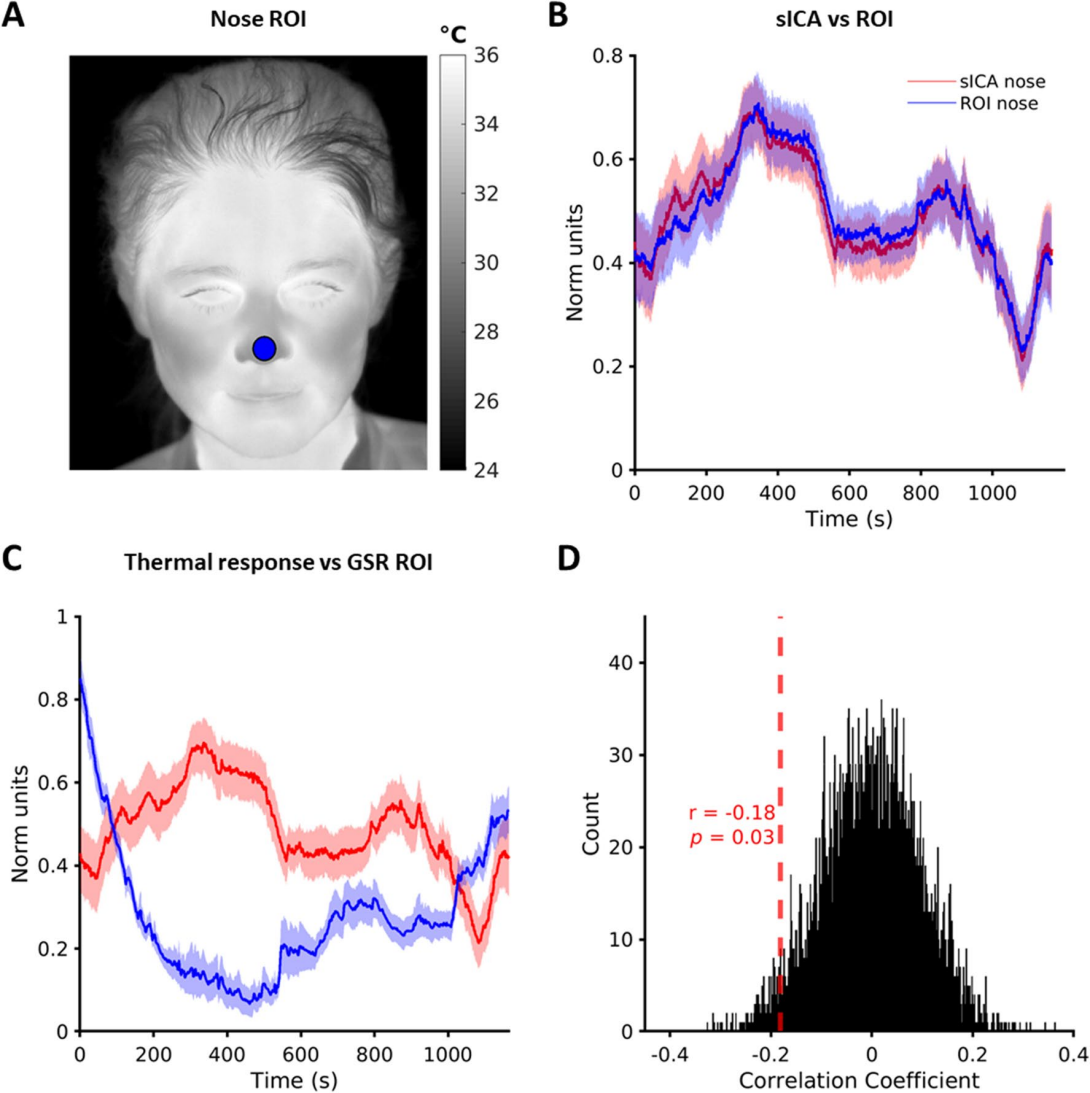
Nose component signal validation by comparison to ROI (region of interest) method and to GSR. (**A**) ROI location on motion-corrected thermal image of a subject (L08) with nine-pixel radius. (**B**) Comparison of group average nose sICA thermal component (red) with that of group average thermal signal obtained by ROI method (blue). (**C**) Group average comparison of thermal response obtained from nose sICA component (red) to GSR (blue). Shading indicates SEM. (**D**) Null distribution obtained with 5000 permutations showing statistical significance of negative correlation between thermal response and GSR.

To validate the ICA-based method, we compared the time courses of the nose IC with those of thermal signals obtained using a ROI located at the nose tip (Fig. 4A). Note that for thermal signal extraction with ROI method, we used the same motion correction algorithm based on optical flow. To our knowledge no other facial thermal imaging study has used an optical flow-based motion correction algorithm. The similarity between signals from the two methods was assessed by evaluating the group average Pearson correlation coefficient of nose IC and the thermal signal extracted from the corresponding ROI of the subject and statistically tested with non-parametric permutation testing (Fig. 4B) (see Methods) (r = 0.94, *p* < 10^–7^, SI Fig. S2 left).

### Inverse relation between nose component signals and GSR

GSR has been extensively validated as an arousal measure in psychophysiological studies^2^. GSR thus provides a valuable benchmark to compare other psychophysiological measures and for facial thermal imaging this has previously been done, albeit for short event-based stimuli^11,39^. We thus compared the time courses of the nose component with that of corresponding GSR signals to further investigate their response relationship during naturalistic emotional experiences. Permutation testing showed a significant inverse relationship (r = − 0.18, *p* = 0.03) (Fig. 4C,D).

### Similarity of physiological changes across subjects

**Figure 5:**
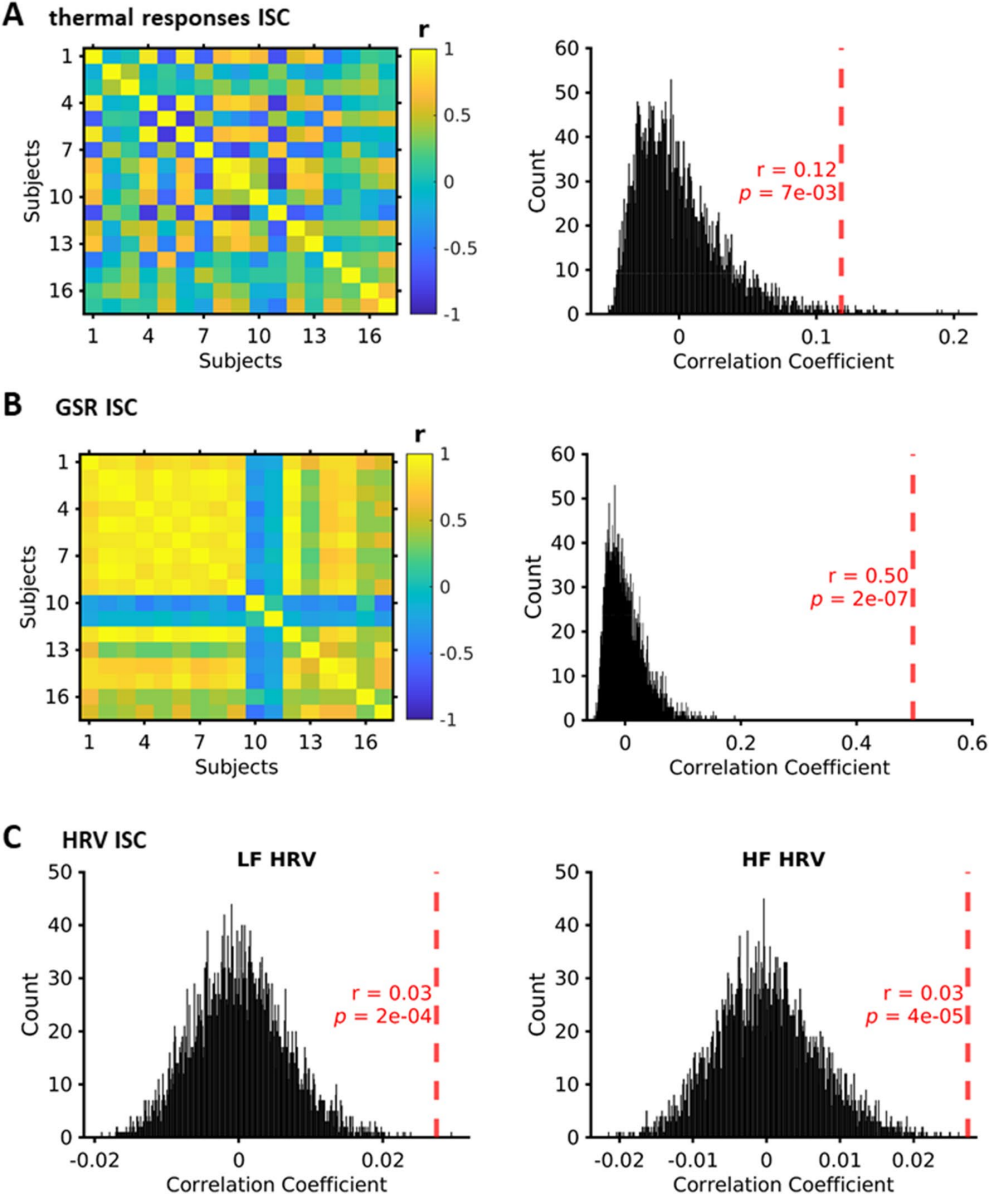
Inter-subject correlation (ISC) analysis. (**A**) correlation matrix of nose component thermal response time series (left) and null distribution obtained with 5000 permutations (right) (see Methods) showing statistical significance of positive mean correlation shown in red. (**B**) correlation matrix of GSR responses curves (left) and right—null distribution obtained with 5000 permutations (right) (see Methods) showing statistical significance of positive mean correlation shown in red (**C**) ISC analysis of low frequency heart rate variability (LF HRV) and its statistical significance (shown in red) (left) across participants when tested with 5000 permutations. ISC analysis for high frequency (HF) HRV (right). FDR corrected for statistical comparisons for LF and HF HRV. Colour bar r denotes Pearson’s correlation coefficient. Histogram r denotes mean correlation coefficient at group level.

We then examined the consistency of all the corresponding physiological variables across subjects using inter-subject correlation (ISC) analysis. We found significant consistency between subjects in the dynamic thermal response patterns for the nose IC (mean r = 0.12, *p* = 0.007, Fig. 5A), and still greater consistency for dynamic GSR (mean r = 0.50, *p* < 0.0001, Fig. 5B). The high ISC of GSR signals seemed to be mostly attributable to the gradual decrease of GSR at the beginning of the movie viewing—by contrast the ISC of GSR signals during the second half of the movie was 0.08 (*p* = 0.03), far lower than the ISC coefficient value for the thermal signal in the second half of the movie of 0.14 (*p* = 0.003). ISC of frequency components of heart rate variability (HRV) also showed significant inter-subject correlation (low frequency HRV: mean r = 0.03, *p*  = 0.0002, Fig. 5C left, high frequency HRV: mean r = 0.03, *p* = 0.00004, Fig. 5C right, p_FDR_ = 0.05).

### Thermal correlates of emotions depicted in the movie

With an exploratory approach, we evaluated whether thermal responses were correlated with the facial emotional scores of the actors in the movie frames. Previous studies, using an event-related design, have found the latency of thermal responses to be around 3.8 s^46^ and 4–5 s^15^ after the stimulus onset. In this study we employed a naturalistic paradigm with continuous narrative instead of discrete events, hence, to compare the emotions of the movie and thermal signals we correlated a 3 s lagged thermal signal with the movie emotion time series. The emotional content of the movie was quantified by computing the scores of facial emotional expressions of the actors in each frame of the movie averaged for each second (see Methods). Thus, each second of the movie was assigned a facial emotional score according to the mean emotion detected from those frames (happiness, sadness, surprise, anger, fear, disgust and contempt), excluding the neutral category (SI Fig. S3 A). A non-parametric permutation test revealed a significant negative correlation between the thermal signal and happiness scores (r = − 0.06, p_FDR_ = 0.0004, SI Fig. S3B) and a significant positive correlation between thermal signal and anger scores (r = 0.05, p_FDR_ = 0.004, SI Fig. S3B). These results are also replicable when using a 4 s lagged thermal signals (happy: r = − 0.06, p_FDR_ = 0.0001, anger r = 0.05, p_FDR_ = 0.004). Furthermore, the results are replicable without introducing lag with (happy: r = 0.06, p_FDR_ = 0.0004; anger: r = 0.05, p_FDR_ = 0.004).

## Discussion

We sought to develop a new data-driven method for facial thermal imaging analysis and introduced novel application of optic flow for motion correction and that of sICA. We validated our method with traditional ROI analysis showing the robustness of our approach. We further demonstrated common physiological responses across subjects as measured by thermal signals, heart rate metrics and GSR, underlining common changes in emotional states induced by the naturalistic stimulus. Crucially these results were obtained in an ecologically valid context.

We restricted our analysis to the nose tip avoiding analysing the cheek and respiratory components since those signals shared what appeared to be noisy respiratory related signatures. The spatial components corresponding to the cheeks were quite broad, and often encroached regions close to lateral aspects of the philtrum. Thus, it is likely that larger regions may have captured respiratory signals in some participants. By contrast, the nose was a distinct and spatially constrained component in all subjects without any overlap of philtrum or cheeks. As a proof-of-concept study, with limited sample size, we wanted to prevent potential multiple comparisons by focusing subsequent analysis on the nose component only. We validated our methodology by comparison to the traditionally employed ROI method and demonstrated remarkably similar results for nose responses with both these methods. The robust correspondence of results with the two methods can be due to state-of-the-art motion correction technique employed. As far as we are aware no other thermal imaging study has utilized optical flow for motion correction of facial thermal imaging. Existing thermal imaging studies employing motion correction have used spatial cross-correlation^12,14^ but these are of limited effectiveness when used with a 3D surface that is not flat, such as the human face. The successful applications of optical flow and spatial ICA in this work thus provide two separate novel methodological contributions, as the former can be used to provide motion correction for ROI based methods as well.

The spatial components of nose and cheeks identified in this study correspond to facial regions widely studied in previous studies^7,11,39,46,47^. The forehead has also shown stimulus induced temperature changes in prior studies^46,48,49^. While some subjects exhibited a distinct forehead spatial component, this was not consistently present in all subjects. In addition to nose and cheeks, a respiration related component was found consistently in all subjects. Respiration monitoring via thermal imaging has already been demonstrated^50,51^. The different facial components were associated with distinct thermal responses. Specifically, the cheek component and respiratory component showed similar time courses and with both being affected by respiration noise. Moreover, the spatial maps of the cheek component showed a diffuse distribution and hence was likely noisier. The successful application of motion correction and spatial ICA in this work demonstrates the utility of this approach for extracting robust thermal signals in longer-time naturalistic paradigms. A promising avenue of future research will be to use spatio-temporal ICA in place of spatial ICA^52,53^ to attempt to separate sympathetic and parasympathetic influences in the same facial regions. Specifically, spatial ICA, as used in the present paper, seeks spatial maps that are maximally independent, so it cannot separate signals that have different causes but manifest in the same facial region, e.g., separate effects of sympathetic and parasympathetic system on blood flow. Temporal ICA separates signals that have independent time courses but has no constraint on spatial maps. Spatiotemporal ICA is a trade-off between these two extremes, so may be useful in separating underlying source signals that are not fully independent in either time or space.

Facial blood flow changes detected by thermal imaging are caused not only by sympathetic (predominantly) but also parasympathetic influences^54,55^. Thus, it is a more complex signal in comparison to GSR which is a uni-dimensional sympathetic response representing arousal^56,57^. Thermal signals thus seem to convey more nuanced representation of sympathetic-parasympathetic balance. The more complex nature of thermal signals may also contribute to greater subject to subject variability and may explain the unexpected anti-correlations between a few subjects (Fig. 5B). GSR signals, due to their reliability, are most widely used in peripheral neurophysiological and psycho-physiological studies, thus providing an optimal benchmark for validation in IRI research^58^. Overall, these results further highlight the biological underpinnings that might make thermal responses useful in differentiating emotional valence.

All the physiological variables (namely thermal imaging, heart rate metrics and GSR) showed significant similarity among subjects, thus further advocating for its use to induce common emotional states. Naturalistic stimuli offer a trade-off between completely uncontrolled stimuli and unconstrained conditions on the one hand (for instance resting state paradigms in neuroimaging), and the strict control of simplistic stimuli (for instance auditory odd-ball paradigm), placing relevant ecological constraints on physiological processes^23^. As emotion is built over a longer narrative, these stimuli can simulate common, everyday emotional experiences and evoke robust and consistent responses in subjects as evident from our inter-subject correlation analysis. These analytical methods of inter-subject correlation analysis, when applied to neuroimaging, have also shown shared neuronal processes underlying emotional states^22,27,41^. Thus, the consistent responses across subjects reported in our study for diverse physiological variables provide convergence of findings on a body level, which at the brain level have already been shown^22,26,40^. This reinforces the wider use of naturalistic paradigms to comprehensively investigate human emotions. In similar vein however, we did not find a temporal consistency of facial expressions among subjects (SI Fig. S4). Facial emotion displays encountered in everyday life situations show high variability^59,60^ and spontaneous expressive behaviour is more complex and ambiguous (Pantic, 2009). This was true in our subjects as well, as they had low emotion expression scores.

## Limitations of the study

Some caveats and limitations of this study should be noted. The three consistent ICA components were identified manually based on visual inspection. An objective automated component selection method can help overcome this limitation in the future^61^. Furthermore, we restricted the number of ICs to fifty, however the spatial components were consistent even when this was increased to eighty (SI Fig. S5). In that case, the bilateral cheek component was often decomposed in separate individual components. Future work could more deeply investigate the spatial patterns of the components, such as their variation across participants, and whether they change in spatial extent (and not just expression) according to different affective responses. Motion correction of facial data has inherent challenges due to the 2D nature of the data while the actual face is a 3D object. We obtained motion correction using 2D dense optical flow, while future work may explicitly map 2D thermal data to a 3D facial model to achieve more precise motion correction. A common standard space for registration of all subjects’ faces will also improve the spatial decomposition. We did not analyse the cheek component signal as the power spectral signature was similar to that of the respiration component. However, we cannot be sure that cheek component signal is devoid of autonomic activation and given these components covered broad areas, we omitted this from analyses. Future studies can delineate these differences. Cross-validation of neuroimaging applications of ISC suggests that to ensure reliability for fMRI data, sample sizes should be N ≥ 20^62^. Two things are worth noting here—first, that (outside of the very strong responses in V1), ISC in fMRI is typically lower than the ISC we observe in thermal responses (compare Fig. 5A with Table 2^62^). The dice coefficient used in Pajula & Tohka encompasses the reliability of all suprathreshold voxels and hence reflects the weaker ISC in e.g. the insula (see their Fig. 1). Second, in fMRI, ISC amongst ICA components is stronger than in voxel space—mirroring the results here. Therefore, while N = 20 is likely a useful rule-of-thumb in fMRI, it is unlikely to generalize exactly to thermal responses when using sICA. We also note that the average sample size of thermal imaging is 17.9 ± SEM of 3.1^4^. Our sample size is consistent with these studies. Nonetheless, and despite conservative statistical correction, although our results are significant, the effect sizes are relatively small. Therefore, as with fMRI, future studies using ISC and thermal responses should use larger samples, particularly if aiming to covary against phenotypic or other independent measures. Future studies could compliment naturalistic stimulus with separately acquired continuous subjective ratings of valence and arousal to further probe temperature perturbations dependence on emotions. Lastly, challenges regarding experimental conditions remain such as the tight experimental control over ambient temperature and humidity.

In sum, we developed a novel data-driven analytical technique for facial thermal imaging analysis and showed robust facial thermal changes evoked by an emotional movie which were correlated with the emotional content of facial expressions in the movie. We also uncovered a physiological consistency among subjects thus signifying common responses elicited by the movie. The effect sizes observed here although modest provide a helpful guide for power analyses in future studies. Future studies could also use simultaneous thermal and brain recordings such as combined facial thermal imaging with functional near infrared spectroscopy^12^ or electroencephalography (EEG), to investigate the brain-body interaction.

## Supplementary Information

Supplementary Information 1.

## Data Availability

Code related to this paper is available from the authors. Data used in the study includes personal identifying facial images of participants, and local ethics approval mandated strict privacy restrictions around their availability outside of the named investigator team. Researchers wishing to access these data will require local ethics approval and a data sharing agreement with QIMR Berghofer.
