# Supplementary material for: Data-driven analysis of facial thermal responses and multimodal physiological consistency among subjects
Source: Sci Rep. 2021 Jun 8;11:12059. doi: 10.1038/s41598-021-91578-5 (PMC8187483; doi:10.1038/s41598-021-91578-5)
Supplement: Supplementary file 1 — Supplementary Information 1. [file 41598_2021_91578_MOESM1_ESM.docx]

Supplementary Information

**Data-driven analysis of facial thermal responses and multimodal physiological consistency among subjects**

Saurabh Sonkusare, Michael Breakspear, Tianji Pang, Vinh Thai Nguyen, Sascha Frydman, Christine Cong Guo, Matthew J. Aburn

**Table S1**

|  | **Questions probing subjective emotion ratings in the questionnaire** | **Ratings** |
| --- | --- | --- |
| Q1  Q2  Q3  Q4 | Is your emotional reaction to the film positive or negative overall?  (***1*** = *extremely negative,* ***4*** *= neutral,* ***7****= extremely positive)*  Overall, was your emotional reaction to the film calm or intense?  (***1*** = *extremely bored/calm,* ***4*** =*neutral,* ***7*** =*extremely*  *excited/intense)*  Which emotion best describes what the film made you feel?  'happy', 'surprise', 'sad', 'disgust', 'fear', ’anger’, 'others'  How strongly did you feel this emotion during the film?  (***1*** = *Not at all,* ***4*** *= mildly* ***7*** *= extremely )* | **Mean = 5.95, SD = 1.43**  **Mean = 5.53, SD = 1.26**  **14-happy, 4-surprise, 1-sad**  **Mean = 5.79, SD = 1.13** |

SD – standard deviation


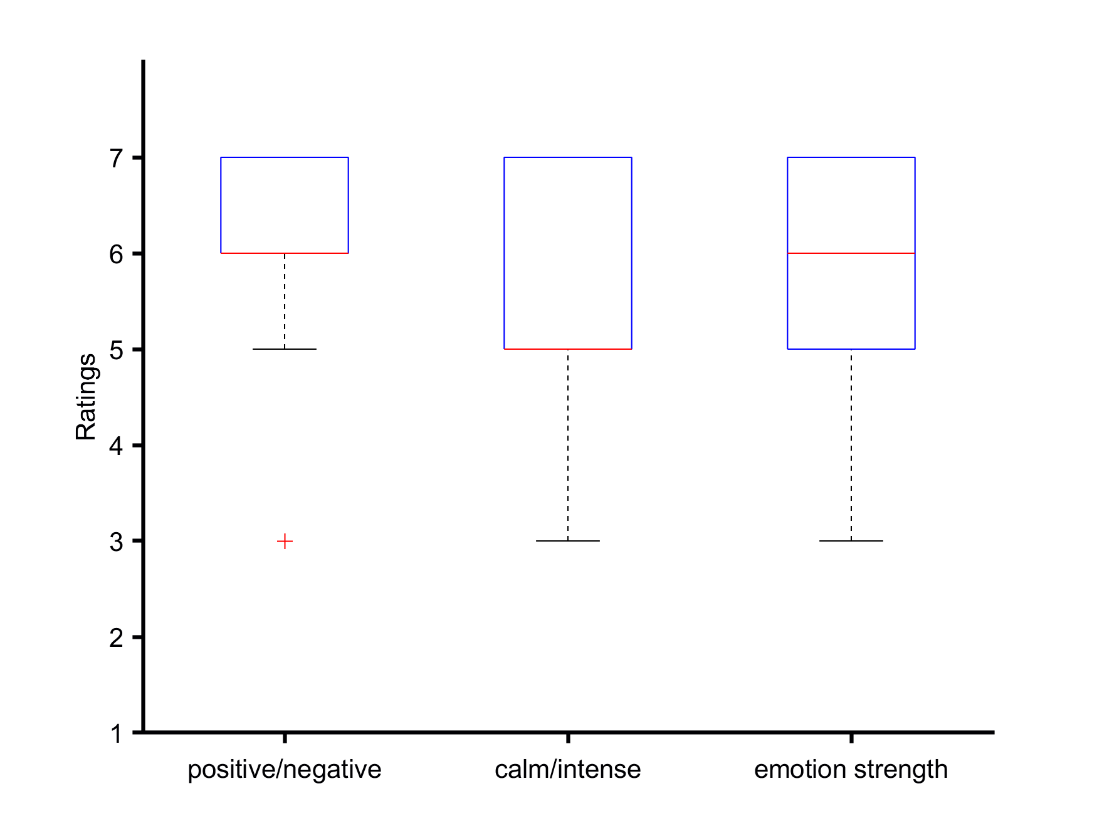


***Figure S1.*** *Emotional ratings distribution.* The three box plots correspond to Q1, Q2 and Q4 in the Table S1. *+* denotes outlier defined as outside 1.5 times the distance between lower (25 percentile) and higher (75^th^ percentile) end of the box.

**Table S2** – Individual subjectwise correlation coefficients between nose component and nose ROI component signals. All correlation coefficients are significant at p<.001 (non-FDR)

| Subject id | Correlation coefficient |
| --- | --- |
| 1 | 0.99 |
| 2 | 0.62 |
| 3 | 0.94 |
| 4 | 0.99 |
| 5 | 0.99 |
| 6 | 0.98 |
| 7 | 0.99 |
| 8 | 0.99 |
| 9 | 0.99 |
| 10 | 0.93 |
| 11 | 0.98 |
| 12 | 0.82 |
| 13 | 0.97 |
| 14 | 0.93 |
| 15 | 0.96 |
| 16 | 0.95 |
| 17 | 0.88 |

**Table S3** - specific parameters inputted in the fastICA code

| [icasig, A, W] = fastica(new_mixedsig,'lastEig', 50, 'approach', 'symm', 'g', 'tanh', 'numOfIC', num,'interactivePCA','on','epsilon',0.0000001); |
| --- |


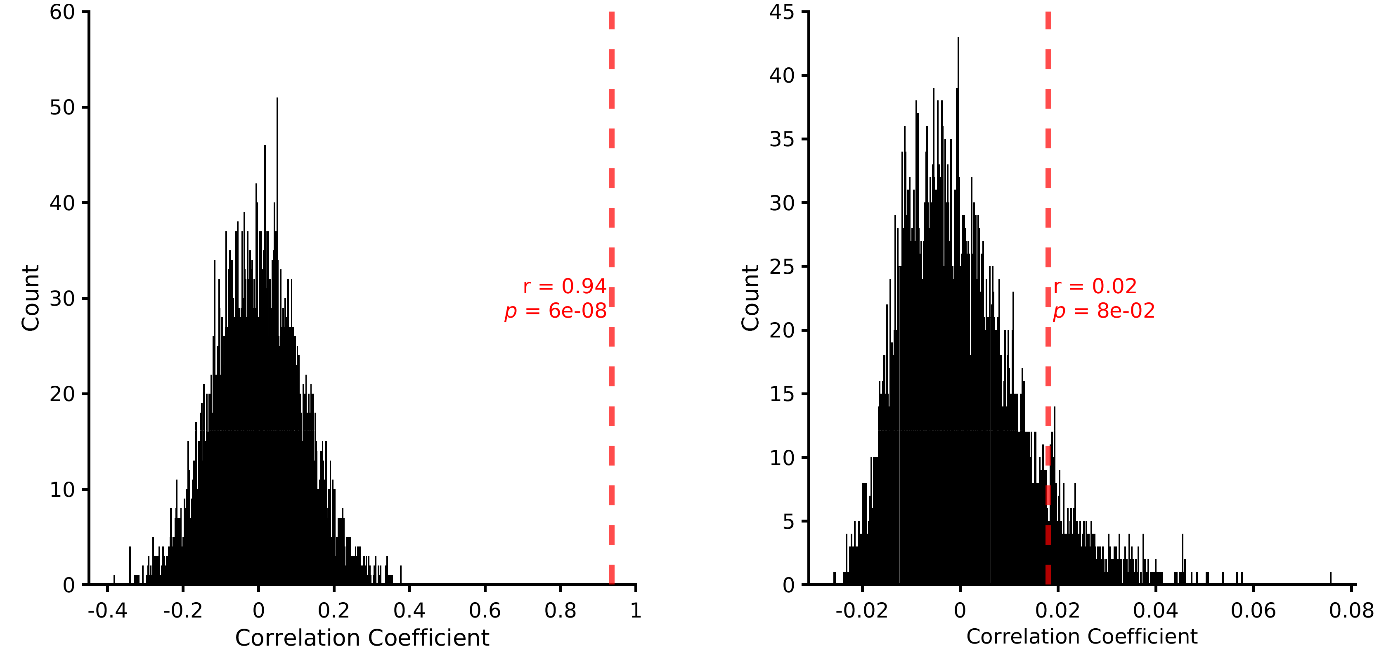


***Figure S2.*** Correlation of sICA nose component with ROI nose signal, and Inter-subject correlation analysis for facial expressions. Left: null distribution obtained with 5000 permutations (see methods) showing statistical significance of positive correlation between nose thermal response from sICA and ROI method, right: null distribution obtained with 5000 permutations (see methods) showing no statistical significance of inter-subject correlation for subjects’ facial expressions.


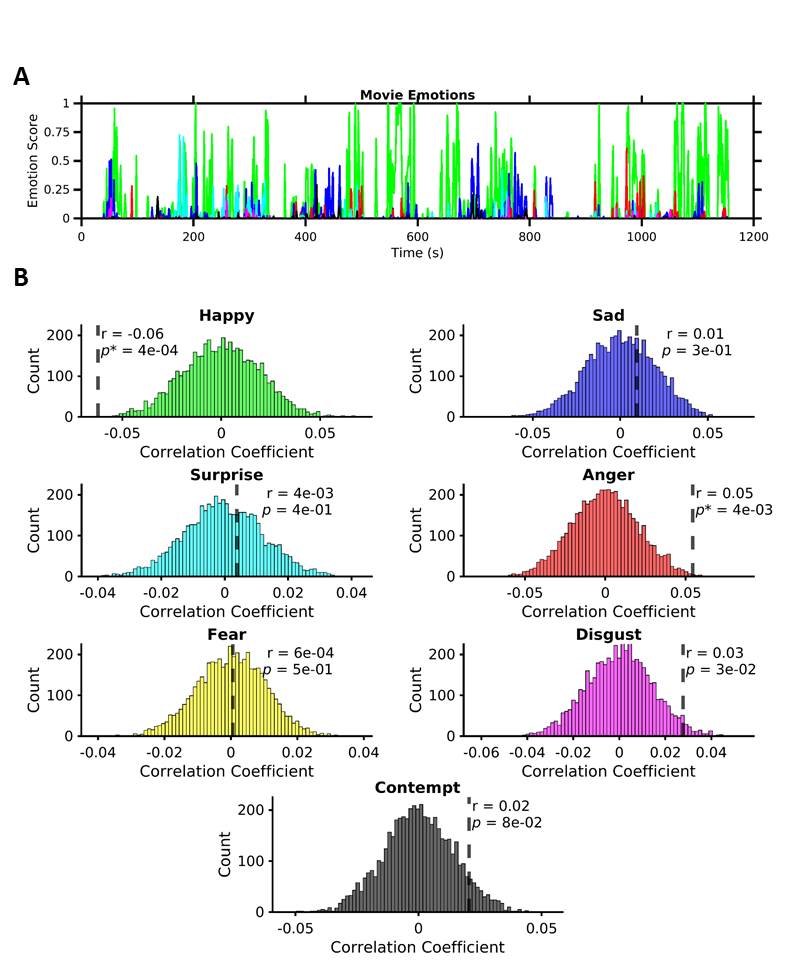
***Figure S3.*** *Thermal correlates of emotion.* **A)** The averaged emotion scores from facial expressions detected in movie frames. **B)** Plots with coloured histograms represent the corresponding emotion category. Histograms were obtained from permutation testing to test statistical significance of correlation between different emotion scores and nose thermal signals. Thermal signal correlation with happy and anger emotion are statistically significant after multiple comparisons (*p** denotes significant results with p_FDR_ < .05). Scientific notation used for r and *p* values where appropriate.





***Figure S4.*** *Facial expression scores of participants for various emotions.* These plots show the mean facial expression scores with shading denoting sem. The emotion scores were predominantly below a score of .1.


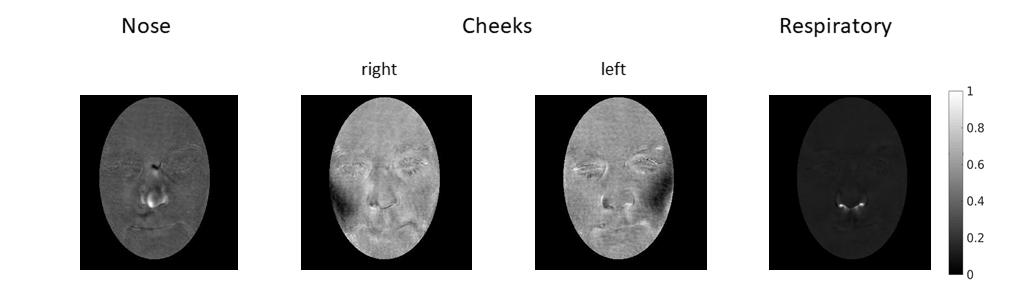


***Figure S5.*** *Spatial components when 80 components were retrieved, instead of 50*. Representative components from one subject (L08) are shown. Note cheek component decomposes into two separate components. Color scale normalized between 0 and 1 for display.
